# Severe gut microbiota dysbiosis caused by malnourishment can be partly restored during 3 weeks of refeeding with fortified corn-soy-blend in a piglet model of childhood malnutrition

**DOI:** 10.1186/s12866-019-1658-5

**Published:** 2019-12-10

**Authors:** Bingfeng Leng, Maria B. Sørensen, Witold Kot, Thomas Thymann, Lukasz Krych, Dennis S. Nielsen

**Affiliations:** 1 Department of Food Science, University of Copenhagen, Rolighedsvej 26, 1958 Frederiksberg C, Denmark; 2 Department of Environmental Science, Aarhus University, Frederiksborgvej 399, 4000 Roskilde, Denmark; 3 Department of Veterinary and Animal Sciences, Section for Comparative Pediatrics and Nutrition, University of Copenhagen, Frederiksberg, Denmark

**Keywords:** Gut microbiota, Malnutrition, Re-feeding diet, 16S rRNA gene amplicon sequencing, Piglets

## Abstract

**Background:**

Childhood malnutrition is a global health challenge associated with multiple adverse consequences, including delayed maturation of the gut microbiota (GM) which might induce long-term immune dysfunction and stunting. To understand GM dynamics during malnutrition and subsequent re-feeding, we used a piglet model with a malnutrition-induced phenotype similar to humans. Piglets were weaned at the age of 4 weeks, fed a nutritionally optimal diet for 1 week post-weaning before being fed a pure maize diet for 7 weeks to induce symptoms of malnutrition. After malnourishment, the piglets were re-fed using different regimes all based on general food aid products, namely Corn-Soy blend (CSB) fortified with phosphorus (CSB+), CSB fortified with phosphorus and skim milk powder (CSB++) and CSB fortified with phosphorus and added whey permeate (CSB + P).

**Results:**

Malnourishment had profound impact on the GM of the piglets leading to a less diverse GM dominated especially by *Akkermansia* spp. as determined by 16S rRNA gene amplicon sequencing. All three re-feeding regimes partly restored GM, leading to a more diverse GM compositionally closer to that of well-nourished piglets. This effect was even more pronounced for CSB++ compared to CSB+ and CSB + P.

**Conclusion:**

The GM of piglets were profoundly disturbed by malnourishment resulting in significantly increased abundance of *Akkermansia* spp. CSB++ may have superior effect on recovering GM diversity compared to the two other food aid products used in this study.

## Background

Malnutrition remains a major challenge for children under the age of 5 years in many developing countries, and causes up to half of all deaths of children (3.1 million) every year [1]. The term malnutrition includes both over- and undernutrition and covers insufficient nutrition including delayed growth as well as symptoms of insufficient or inappropriate intake of protein, essential fatty acids, vitamins and minerals [2]. Although current therapeutic approaches, such as ready-to-use therapeutic food (RUTF), have reduced the overall mortality [3], these treatments still have limited efficacy in terms of ameliorating persistent sequelae, notably stunting, wasting, and immune dysfunction [4]. Further, children who had been successfully treated with RUTF for moderate acute malnutrition have been found to be still at increased risk of malnutrition and death in the years following recovery [5].

Food insecurity, infections, age and geography are considered major risk factors for malnutrition. Recently gut microbiota (GM) dysbiosis and immaturity have been strongly associated with certain forms of malnutrition [6, 7]. The GM of severely malnourished children is associated with increased relative abundance of Proteobacteria (including pathogenic taxa), but are also characterised by having a GM immature for age, with reduced relative abundance of several *Bifidobacterium* and *Lactobacillus* spp. as well as obligate anaerobic short chain fatty acid producing taxa [8, 9]. Blanton et al. demonstrated that transplanting a GM immature for age from malnourished Malawian children into germ-free mice also transferred the malnourished phenotype to the mice showing impaired growth [10]. The mice receiving GM from malnourished children also had impaired lean body mass gain, and metabolic changes in liver and muscle, which was consistent with previous studies using piglets as a model for malnourished children, where the same patterns have been observed [11–13]. The GM immaturity for age observed in severely malnourished children has been found to persist even after therapeutic food intervention [9], indicating that GM dysbiosis is alleviated slower than the phenotype. This could possibly also explain the higher risk of complications (e.g. relapse into a malnourished state) the first year following refeeding after malnutrition [5], as it has also been found for other disease phenotypes, where GM plays a role in disease etiology [14]. All together these findings strongly indicate that malnutrition is linked to GM dysbiosis which in turn influences the chance of successful re-feeding, with potential long-term consequences for the host.

A number of studies have evaluated the clinical potential of RUTF [15, 16]. Whereas corn-soya blends, with or without added dairy products, is the most commonly used RUTF, the possible links between malnutrition, GM and different formulations of RUTF remain poorly understood. Using piglets as a model for malnourished children, the aim of the present study was to investigate how GM composition changes in response to malnutrition (exclusive maize diet) and subsequent different re-feeding strategies consisting of Corn Soya Blend (CSB) fortified with either phosphorus (CSB+), phosphorus and skim milk powder (CSB++), or with phosphorus and added whey permeate (CSB + P). These specific diets were chosen as they represent clinically relevant therapeutic diets for re-feeding. The extra phosphorus in all three refeeding diets was in the shape of mono-calcium-phosphate (CSB+), skim milk powder (CSB++) or whey permeate (CSB + P). Whereas the purpose of this was to avoid re-feeding hypophosphatemia, i.e. a common clinical complication during nutritional rehabilitation, it is not known if these three diets would affect the gut microbiome differently. The results may give a reference to evaluate the effectiveness of re-feeding-diets on restoring the GM composition, which in turn might help to prevent the recurrent malnutrition.

## Methods

### Animals, experimental design and diets

All animal experimental procedures were approved by the Danish Animal Experiments Inspectorate. A total of 73 female piglets (Landrace x Yorkshire x Duroc, Maglegaard I/S) purchased from a commercial pig production site were included in this study [13]. Housing conditions and animal handling were as previously described [13]. At the age of 4 weeks all piglets were weaned to a nutritionally optimised diet (Table 1) and fed for 1 week in order to adapt to the new environment. Afterwards, 6 piglets were sacrificed (Reference, *n* = 6) to determine GM composition prior to the experimental period. Eleven piglets remained on the nutritionally optimal reference diet, while 56 piglets were switched and given ad libitum access to a diet consisting of pure maize for 7 weeks. Pure maize was chosen as it represents a typical household diet in developing countries, and leads to a degree of malnutrition. After 7 weeks, the 11 piglets fed with optimized reference diet (Reference7, *n* = 11), together with 15 piglets fed with maize (Maize7, *n* = 15) were sacrificed. The remaining piglets continued either on the maize diet for 3 weeks (10 weeks in total, Maize10, n = 15), or were allocated to a re-feeding diet consisting of either CSB+ (*n* = 9), CSB + P (n = 9), or CSB++ (*n* = 8) for 3 weeks and then sacrificed. All CSB diets represented clinically relevant therapeutic diets, and were produced according to the World Food Programme (WFP) specifications for food aid commodities at the time of production (Michiels Fabrieken, Zulte, Belgium) as previously lined out [11]. All piglets were given ad libitum access to their respective diets during the experiment. The nutrient composition of the diets is shown in Table 1. For sampling the pigs were first anaesthesized using a combination of zolazepam, tiletamin, xylacin, buthorphanol and ketamine, injected intramuscularly at 1 ml/10 kg. When full anaesthesia was achieved, the pigs were euthanized with an intracardiac injection of sodium pentobarbitone. Colon content collected after slaughtering was snap-frozen in liquid nitrogen and stored at − 80 °C prior to further analysis.

**Table 1:** Diet composition of the different feed types used (n.d., non detected)^a^

		Reference	Maize	CSB+	CSB + P	CSB++
Energy	MJ/kg	8.71	9.37	15.75	15.67	16.9
Protein	g/kg	219	90	199	193	147
Carbohydrate	g/kg	526	715	686	690	522
Fat	g/kg	61.2	43	99.5	92	89.1
Starch	g/kg	366	607	539	495	385
Sucrose	g/kg	42.9	17.6	13.9	18.8	101.3
Lactose	g/kg	23.4	n.d.	n.d.	n.d.	40.5
Vitamin A	IE/kg	12	n.d.	20	26	18
Vitamin D3	IE/kg	1	n.d.	0.4	0.36	0.41
Vitamin E	mg/kg	220	n.d.	31	29	3.48
Biotin	mg/kg	0.24	n.d.	0.21	0.2	13.07
Pantothenic acid	mg/kg	28	n.d.	74	71	73
Vitamin B1	mg/kg	2.4	n.d.	4.4	4.4	3.2
Vitamin B2	mg/kg	10	n.d.	7.6	8.7	7.2
Vitamin B6	mg/kg	7.2	n.d.	18.5	17.2	18.2
Vitamin B12	mg/kg	0.02	n.d.	0.02	1.9	0.05
Niacin	mg/kg	24	n.d.	54	49	52
Vitamin K3	mg/kg	4.4	n.d.	2.7	2.4	1.6
Calcium	g/kg	10.1	0.43	5.6	4.8	6.3
Phosphorus	g/kg	7.66	3.3	4.8	4.8	5.2
Sodium	g/kg	1.88	0.1	1.59	2.31	1.92
Magnesium	g/kg	1.63	1.15	1.71	2.99	1.17
Potassium	g/kg	10	3.52	12.8	12.02	12.4
Chloride	g/kg	1.59	n.d.	1.5	1.35	1.5
Iron	mg/kg	293	29.6	144	132	111
Manganese	mg/kg	66.2	n.d.	7.12	6.52	3.68
Copper	mg/kg	166	n.d.	0.39	0.36	0.28
Zinc	mg/kg	155	n.d.	53.56	49.86	55.62
Iodine	mg/kg	0.3	n.d.	0.4	0.36	6.1
Selenium	mg/kg	0.3	n.d.	0.03	0.03	1.1

^a^The Reference diet was formulated from corn, soya, fish meal, skim milk powder, palm oil, amino acids, vitamins and minerals. The Maize diet was formulated as pure maize with no added supplements. All CSB diets were based on a base of corn soya blend, with added monocalciumphosphate (CSB+), mono-calciumphosphate and skim milk powder (CSB++) or mono-calciumphosphate and whey permeate (CSB + P)

### Assessment of degree of malnutrition

Assessments of the degrees of underweight (weight relative to age), stunting (length relative to age) and wasting (weight relative to length) following 7 weeks of nutritional depletion on the maize diet were derived from the Waterlow classification used for children [17] and carried out as described previously [11].

### DNA extraction

Microbial genomic DNA from the colon content was extracted using MO BIO PowerSoil® DNA Isolation Kit (MO BIO Laboratories Inc., Carlsbad, CA, USA) following the manufacturer’s protocol but with the addition of an initial bead-beating step (FastPrep, 3 × 6.5 M/s for 15 s) for improved cell lysis. DNA concentration and purity were verified using the Nanodrop 2000 (Thermo Scientific, Denmark). Extracted DNA was stored shortly at − 20° for up to 2 weeks before being moved to − 60° until further analysis.

### Tag-encoded 16S rRNA gene amplicon high throughput sequencing

The GM profiles were determined using tag-encoded 16S rRNA gene amplicon MiSeq (Illumina) based high throughput sequencing. Two rounds of PCR were performed to prepare libraries for amplicon sequencing. Amplicons (~ 460 bp) including the V3-V4 and V3 regions of the 16S rRNA gene were amplified, tagged and sequenced as described previously [18]. Sequencing was performed using 2x250bp, V2 chemistry kit for MiSeq (Illumina) high throughput sequencing.

The raw dataset containing pair-ended reads with corresponding quality scores were merged and trimmed, purged for chimeric reads and de novo Operational Taxonomic Units (OTU) determined as previously lined out [19, 20]. The Green Genes (13.8) 16S rRNA gene collection was used as a reference database [21]. The Quantitative Insight Into Microbial Ecology (QIIME) open source software package (1.8.0 and 1.9.1) [22] was used for analysis. After quality control and removing chimeric reads a total of 2,173,891 reads were generated from all colon content samples, with an average of 29,377 sequences per sample (min = 6354; max = 117,557; SD = 23,092).

Alpha and beta diversity analysis were performed as previously described using iterative subsampling (6000 reads/sample) [22]. Permanova (compare_categories.py, Qiime 1.8.0) and PermanovaG (the Generalized UniFrac R package; GUniFrac) [23] were used to evaluate group differences using weighted, unweighted and generalized UniFrac distance matrices, respectively [19, 24]. All distance matrices were generated based on rarefied (6000 reads/sample) OTU tables. The G test of independence (q_test) and ANOVA were used to determined qualitative (presence/absence) and quantitative (relative abundance) associations of OTUs with given categories, respectively [19]. The Parametric Student’s t-test (QIIME v9.1, 6000 reads/sample, *p*-values FDR-corrected) was used to test frequencies of species level OTUs between groups fed different diets [19].

### Absolute quantification of *Akkermansia muciniphila* by qPCR

The relative abundance of *Akkermansia muciniphila* was determined by qPCR using the 7500 Fast Real-time PCR System (Applied Biosystems, CA, USA). Two primer sets, universal F_338 (5′-ACWCCTACGGGWGGCAGCAG-3′), R_518 (5′-ATTACCGCGGCTGCTGG-3), and *A. muciniphila* specific AM2 (5′- CCTTGCGGTTGGCTTCAGAT-3′) and AM1 (5′-CAGCACGTGAAGGTGGGGAC-3′) (9) targeting the 16S gene, were used. The reaction mix (20 μl) consisted of: 0.5 μl of each universal or *A. muciniphila* specific primer set, 10 μl of Fast SYBR® Green Master Mix (Applied Biosystems), 4 μl of sterile MilliQ water and 5 μl of genomic DNA (~ 20 ng/μl). The temperature profile for thermocycling was as follows: 95 °C for 5 min, followed by 40 cycles of 95 °C for 15 s, and 60 °C for 1 min. The fluorescence acquiring was set in the annealing/extension step. Following the amplification, a melting curve analysis was performed to distinguish an eventual nonspecific amplification. Standard curves were generated using 10-fold serial dilutions of *A. muciniphila* pure culture (DSMZ 22959) and *Escherichia coli* K12 genomic DNA of known concentrations. Negative controls (sterile MilliQ water) were included in all runs.

### Goblet cells evaluation

Tissue sections from the colon were collected and fixed in paraformaldehyde. Following dehydration in ethanol the samples were embedded in paraffin and sliced into 2–4-μm cross sections. To visualize mucin-filled goblet cells, the slices were stained with Alcian blue (at pH 2.5) and periodic acid-Shiff. The goblet cell area relative to total mucosal area within a reference space was estimated using the Visiopharm software package (Visiopharm, Hørsholm, Denmark).

### Availability of data

The 16S rRNA gene amplicon sequence dataset has been uploaded to the European Nucleotide Archive under accession number PRJEB33018. Other datasets analysed during the current study are available from the corresponding author on reasonable request.

## Results

### Effect of malnutrition and re-feeding on gut microbiota composition

After 7 weeks of feeding, all piglets fed maize were severely malnourished compared with the reference group, and the short term refeeding no matter with CSB+, CSB++ or CSB + P did not restore the growth. The weight increment was limited (12–13 g/kg per day) and equal between the refeeding groups [11].

Alpha diversity analyses of the colon microbial community showed that malnourished piglets had lower diversity compared with well-nourished piglets (Additional file 1: Figure S1). The number of observed species was significantly lower for piglets fed with maize for 7 and 10 weeks compared to that of piglets fed with optimized reference diet for 7 weeks, while the Shannon Diversity Index was not different between these two groups. During refeeding there was a numeric, but non-significant trend, that piglets re-fed with CSB+, CSB++ and CSB + P harboured a higher number of observed species than those fed with maize for 7 and 10 weeks (Table 2).

**Table 2:** Observed species and Shannon diversity as influenced by feeding regime

Category (n)^*^	Relative age (weeks)	Observed species average (SD)	Shannon Diversity (SD)
Reference (6)	0	107 (8.9)^ab^	4.9 (0.5)^a^
Reference 7 weeks (11)	7	111 (10.7)^a^	4.9 (0.6) ^a^
Maize 7 weeks (15)	7	91 (17.9)^ab^	3.0 (1.6) ^a^
Maize 10 weeks (15)	10	83 (21.1)^b^	2.1 (1.4) ^a^
CSB+ (9)	10	100 (16.1)^ab^	3.6 (1.3) ^a^
CSB + Permeate (9)	10	100 (20.3)^ab^	4.0 (1.4) ^a^
CSB++ (8)	10	111 (13.9)^ab^	4.7 (0.4) ^a^

^*^ The Reference diet was formulated from corn, soya, fish meal, skim milk powder, palm oil, amino acids, vitamins and minerals. The Maize diet was formulated as pure maize with no added supplements. All CSB diets were based on a base of corn soya blend, with added monocalciumphosphate (CSB+), mono-calciumphosphate and skim milk powder (CSB++) or mono-calciumphosphate and whey permeate (CSB + P)

Piglets were fed either the Reference diet for 7 weeks or a maize-based diet for 7 weeks followed by refeeding with one of the 3 differents (CSB+, CSB + Permeate or CSB++). Values sharing same superscript-letter are not significantly different (*p* > 0.05), otherwise *p* < 0.05 (as determined by a nonparametric t-test method, Monte Carlo simulation, 999 permutations). SD, standard deviation

**Fig. 1:**
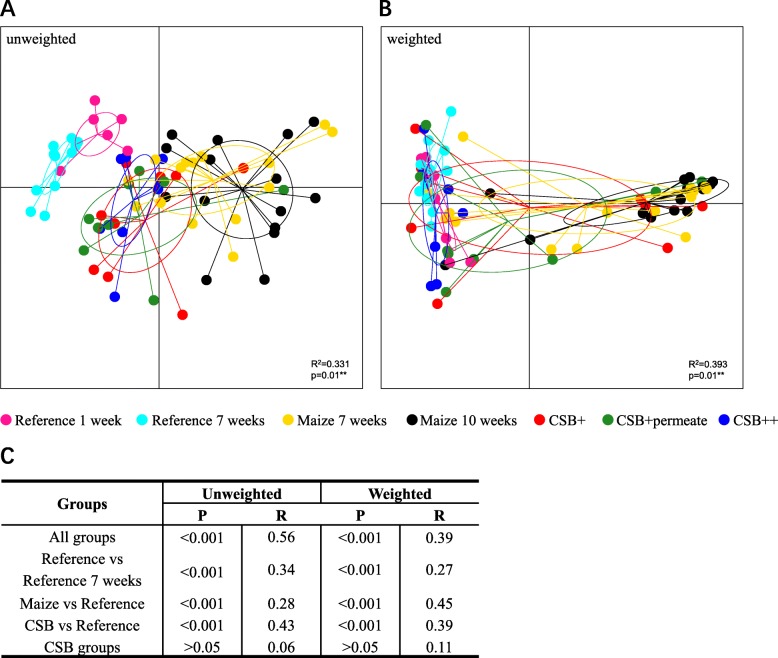
PCoA plot based on (**a**) unweighted and (**b**) weighted UniFrac distance matrices of 16S rRNA gene derived amplicons, and group significance of beta diversity between different diet groups (Adonis) (**c**). Both the unweighted (**a**) and weighted (**b**) UniFrac distance matrices showed clustering according to group (**c**). The degree of variation among 10 jackknifed replicates of PCoA is showed as confidence ellipsoids around each group

Unweighted and weighted UniFrac distance matrices (Fig. 1) showed a clear separation according to diet. The GM of well-nourished piglets clearly clustered away from malnourished piglets. Refeeding influenced GM composition with refed piglets showing a clear pattern of becoming more similar to the well-nourished, reference pigs (Fig. 1b and c). This trend was especially pronounced for the piglets refed using CSB++ (Fig. 1).

### Phyla and genera abundance and distribution

**Fig 2:**
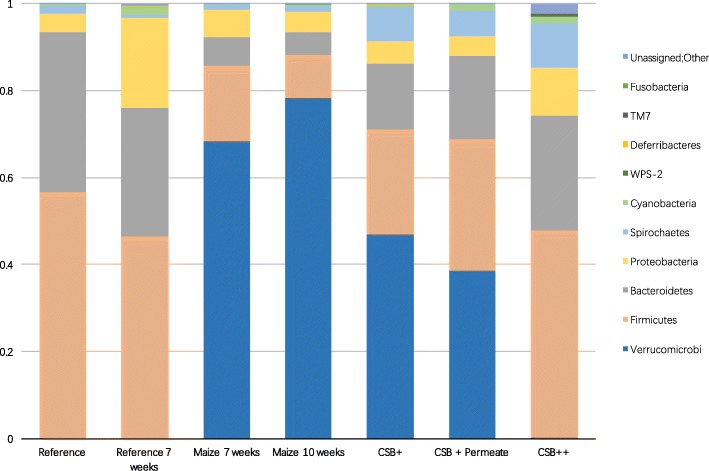
Colonic microbiota composition at phylum level. The average phylum-level relative abundance and distribution as determined by 16S rRNA gene V3 V4-amplicon sequencing of colon content samples piglets fed with reference diet for 1 week (reference), then fed with either reference diet or maize for 7 weeks, and lastly fed with either maize, CSB+, CSB + Permeate or CSB++ for 3 weeks

**Fig. 3:**
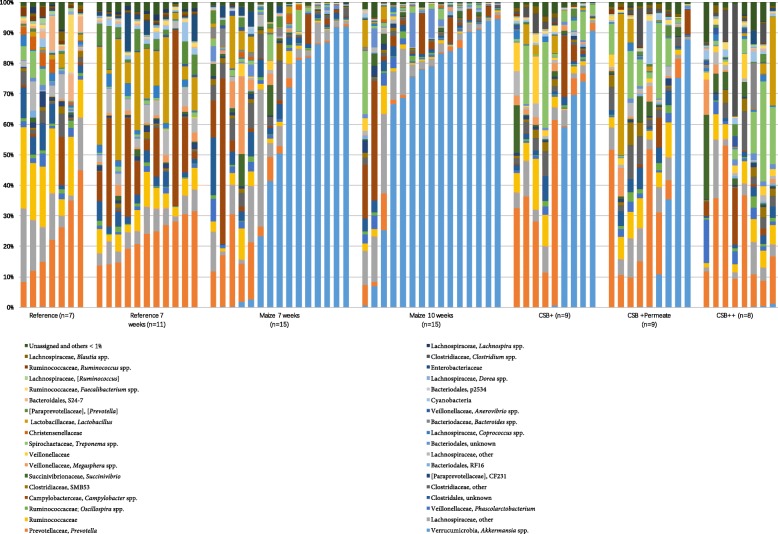
Colonic microbiota composition at genus level. The relative abundance and distribution of genera as determined by 16S rRNA V3 V4-region amplicon sequencing of colon content samples from piglets fed with reference diet for 1 week (reference), then fed with either reference diet or maize for 7 weeks, and lastly fed with either maize, CSB+, CSB + Permeate or CSB++ for 3 weeks

The diversity and community level differences between different diet groups as well as the effect of refeeding were also reflected in the relative abundance of several OTUs being significantly different between the groups (Fig. 2 and Fig. 3). In both reference diet groups (Reference7 and Reference10), the predominant phyla in the colon microbiome were Firmicutes and Bacteroidetes, with the only larger difference between the 7 and the 10 weeks reference groups being decreased abundance of Firmicutes and Bacteroidetes, but increased abundance of Proteobacteria (*p* < 0.05) in the week 10 group. Malnutrition for both 7 and 10 weeks led to a surprisingly high relative abundance of Verrucomicrobia, represented by a single OTU. This OTU was also detected in low numbers in the well-nourished piglets of the same age. Interestingly, during refeeding the abundance of Verrucomicrobia tended to decrease, especially in the CSB++ group (CSB++ vs. CSB+ and CSB + P, *p* < 0.05), where Verrucomicrobia after 3 weeks of refeeding was almost as low as in both reference groups.

Phylum Verrucomicrobia was represented by a single species, *Akkermansia muciniphila* (Fig. 3), dominating the GM of the malnourished piglets. In contrast to the abundance of *A. muciniphila*, the abundance of *Prevotella* spp. was low in Maize 7 weeks (3.56%) and Maize 10 weeks (1.06%) and higher in CSB+ (12.85%), CSB + P (17.13%) and CSB++ (21.22%) (p < 0.05). Similarly, the relative abundance of other genera, such as *Faecalibacterium*, *Bulleidia*, and *Ruminococcus* was lower in the malnourished piglets (Maize groups) and higher in CSB groups and the reference groups (p < 0.001).

The high relative abundance of *A. muciniphila* in the malnourished piglets was further verified with qPCR. Indeed, qPCR analysis confirmed that malnutrition did indeed increase the relative abundance of *A. muciniphila*, but not as much as suggested by high throughput amplicon sequencing of the V3-V4 region of the 16S rRNA gene (Additional file 2: Figure S2). Suspecting that primers targeting region V3-V4 could favour *A. muciniphila* amplification all samples were sequenced using primers targeting the V3-region of the 16S rRNA gene as well [20]. Sequencing of the V3-region confirmed *A. muciniphila* was one of the predominating phyla in piglets fed with maize for 10 weeks, though the relative abundance of *A. muciniphila* as determined by 16S rRNA gene V3-region sequencing was lower than the relative abundance of *A. muciniphila* when targeting the V3-V4 region. Importantly, all three methods (qPCR, V3-V4 or V3 region amplicon sequencing) showed that malnourishment led to an increased abundance of *A. muciniphila* and that refeeding with fortified CSB, especially CSB++, led to a decrease in the relative abundance of *A. muciniphila* to levels near what was observed in the well-nourished piglets (Additional file 3: Figure S3).

## Discussion

As a human-sized omnivorous species with an anatomy, physiology and metabolism comparable to humans, the pig has been extensively used as a model in nutritional studies [25]. From a GM perspective several recent studies have shown that there are pronounced differences in the composition of the most abundant genera in the human and the piglet gut microbiome, but also that the human and pig GM show pronounced overlap at the gene functional level [26, 27] underlining the potential of using pigs for biomedical research [28]. Moreover, the high similarities between humans and pigs regarding the dynamics of postnatal maturation of GM diversity and structure has made the pig a useful model for human infants, where it has been applied to study e.g. nutritional interventions [28].

In agreement with previous studies, our data showed that the dominant phyla in the GM of well-nourished piglets were Firmicutes, Bacteroidetes and Proteobacteria [29]. However, when the piglets underwent severe malnutrition using a nutritionally suboptimal pure maize diet, a notable decrease was seen in both GM diversity (as determined by number of observed species) and composition compared with well-nourished piglets. A similar pattern has been observed among humans during malnutrition or caloric restriction [9]. After prolonged maize feeding, the number of observed species were significantly lower than in the piglets fed with the reference (optimal) diet (Monte Carlo, *p* < 0.05). Interestingly, it was observed that the GM of malnourished piglets became dominated by the mucin degrading bacteria *A. muciniphila* and that the proportion of this bacterium was further increased by longer maize feeding.

Refeeding with CSB diets increased the GM diversity and changed the GM composition. In both groups re-fed with CSB+ or CSB + P, there is a clear tendency that Verrucomicrobia relative abundance decreases with refeeding, though it still remained more abundant than in the control groups after 3 weeks of refeeding. In piglets re-fed with CSB++ the relative abundance of Verrucomicrobia decreased to levels (0.3%) comparable to well-nourished piglets. Previously, Hother et al. (2017) have reported that all 3 CSB formulations were equally effective at promoting growth of piglets during a 3 weeks re-feeding, but with a limited rate of 12–13 g/kg per day [11]. Based on the present study, each of the CSB formulations differently influenced piglet GM, with the CSB++ showing higher ability to restore the GM of piglets towards the ‘normal’ composition of well-nourished piglets. Whether the GM differences observed after refeeding also influence the ability to resist further episodes of malnutrition still remains to be investigated. The underlying reasons why CSB++ is more efficient in restoring the GM of malnourished piglets remains to be investigated, but it can be speculated that the the milk proteins that are present in CSB++ but not CSB+ or CSB + P, may play a pivotal role.

The relative abundance of *Akkermansia muciniphila* determined by 16S rRNA gene amplicon sequencing (V3-V4 region) was surprisingly high in the GM of malnourished piglets. We thus determined the relative abundance of this species using specific qPCR, which confirmed the higher abundance of *Akkermansia* in the GM of these piglets, though the relative abundance of *Akkermansia* spp. determined by qPCR was lower than the values determined by amplicon sequencing. We thus re-sequenced these samples using different primers targeting the V3 region. Sequencing of the V3 region revealed that *Akkermansia* spp. relative abundance was closer to the results generated with qPCR. It is a well-described phenomenon that choice of primers and target-region influence the outcome of amplicon-sequencing approaches [30], but no matter the method *Akkermansia* spp. was among the dominant taxa in the malnourished piglets – and in all cases all three methods consistently confirmed that refeeding leads to lower *Akkermansia* spp. relative abundance and that compared with CSB+ and CSB + P re-feeding with of CSB++ efficiently decrease the abundance of *A. muciniphila* in the GM of piglets, even within a the relatively short period of 3 weeks, and thus may help to restore GM composition.

*Akkermansia* is an abundant inhabitant of the intestinal tract of humans and many other animals*.* Several studies have shown that *A. muciniphila* is associated with protection against metabolic syndrome, obesity and type 2 diabetes and other diseases [31, 32]. As a mucin degrading bacteria, it can provide propionic and acetic acid to benefit other bacteria in or near the mucosa, and promote mucus secretion, making the epithelial barrier more robust [33]. In this study, we investigated further how the dominance of *Akkermansia* spp. in the malnourished piglets associates with goblet cell density in the mucosa in the colon. Malnourished piglets showed less goblet cells (Additional file 4: Figure S4), indicating either lower number of cells or increased emptying of the goblet cells increasing mucin secretion. Consequently, the increased abundance of *Akkermansia* spp. could simply reflect increased substrate (mucin) abundance. Whether it is the malnutrition per se or other factors that induce emptying of the goblet cells remains to be investigated.

## Conclusions

Consistent with previous human studies, a profound effect of malnutrition on GM composition was observed. The influence of different RUTF on GM after refeeding was also evaluated. The feed CSB++ fortified with skimmed milk seems most efficient in restoring the GM in malnourished piglets compared with low-cost CSB+ and CSB+ fortified with whey permeate raising questions about the optimal trade-off between the price of RUTF and its effect on restoring host health including GM biosis.

## Supplementary information

**Additional file 1: Figure S1**. Rarefaction curves based on OTUs. The rarefaction curve of observed species detected in colon content samples from piglets fed with reference diet for 1 week (reference), then fed with either reference diet or maize for 7 weeks, and lastly fed with either maize, CSB+, CSB + Permeate or CSB++ for 3 weeks.

**Additional file 2: Figure S2**. Relative abundance of *Akkermansia muciniphila* was determined by qPCR (determined as number of *A. muciniphila* 16S rRNA gene copies relative to the total number of 16S rRNA gene copies pr. sample).

**Additional file 3: Figure S3**. The relative abundance and distribution of genera detected by targeting V3 V4 region in colon content samples from piglets fed with reference diet for 1 week (reference), then fed with either reference diet or maize for 7 weeks, and lastly fed with either maize, CSB+, CSB + Permeate or CSB++ for 3 weeks.

**Additional file 4: Figure S4**. The ratio of goblet cell area to total tissue area of colon tissue from the reference, well-nourished piglets and the malnourished piglets ere determined by Alcian blue staining.

## Data Availability

The 16S rRNA gene amplicon sequence dataset has been uploaded to the European Nucleotide Archive under accession number PRJEB33018. Other datasets analysed during the current study are available from the corresponding author on reasonable request.

## Abbreviations

CSB: Corn-Soy blend
CSB+: CSB fortified with phosphorus
CSB++: CSB fortified with phosphorus and skim milk powder
CSB+P: CSB fortified with phosphorus and added whey permeate
GM: Gut microbiota
RUTF: Ready-to-use therapeutic food
SAM: Severe acute malnutrition
