# Supplementary figures and images for: Severe gut microbiota dysbiosis caused by malnourishment can be partly restored during 3 weeks of refeeding with fortified corn-soy-blend in a piglet model of childhood malnutrition
Source: BMC Microbiol. 2019 Dec 10;19:277. doi: 10.1186/s12866-019-1658-5 (PMC6902335; doi:10.1186/s12866-019-1658-5)

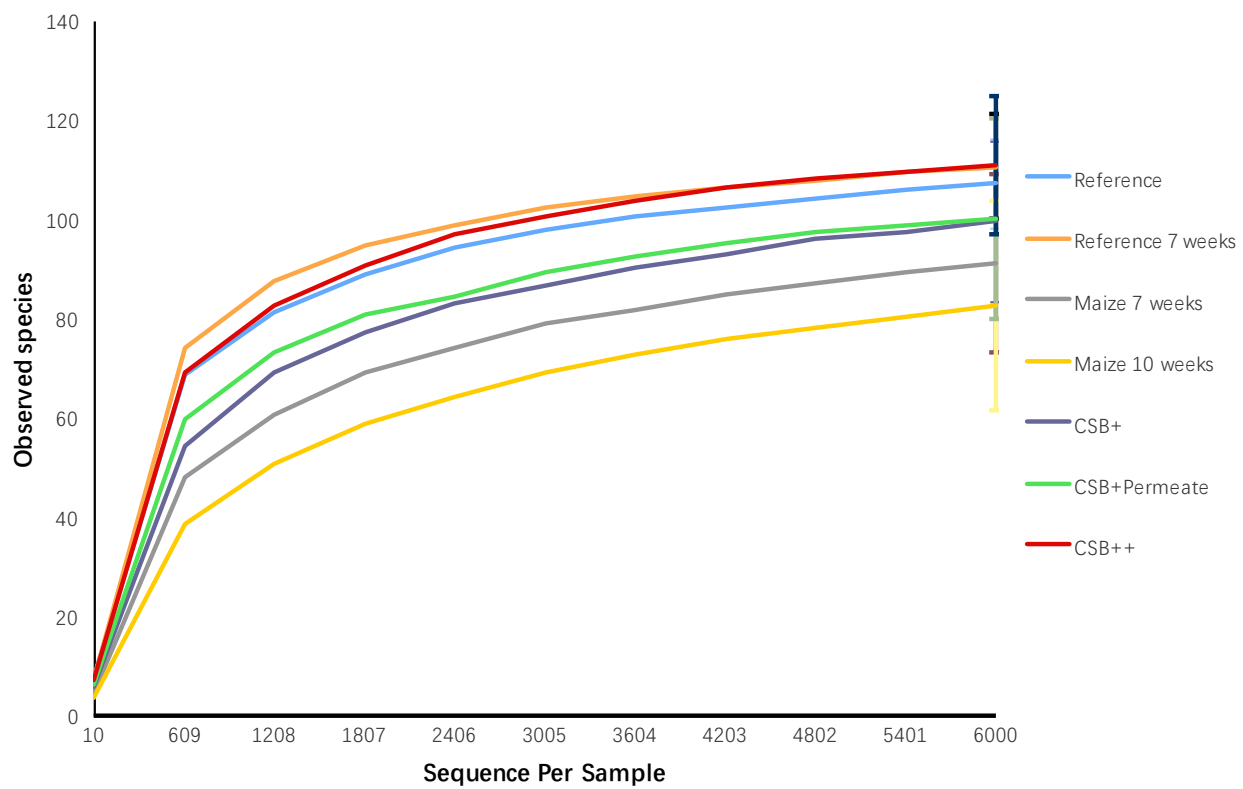

Supplement: Supplementary file 1 — Additional file 1: Figure S1. Rarefaction curves based on OTUs. The rarefaction curve of observed species detected in colon content samples from piglets fed with reference diet for 1 week (reference), then fed with either reference diet or maize for 7 weeks, and lastly fed with either maize, CSB+, CSB + Permeate or CSB++ for 3 weeks. [file 12866_2019_1658_MOESM1_ESM.pdf]

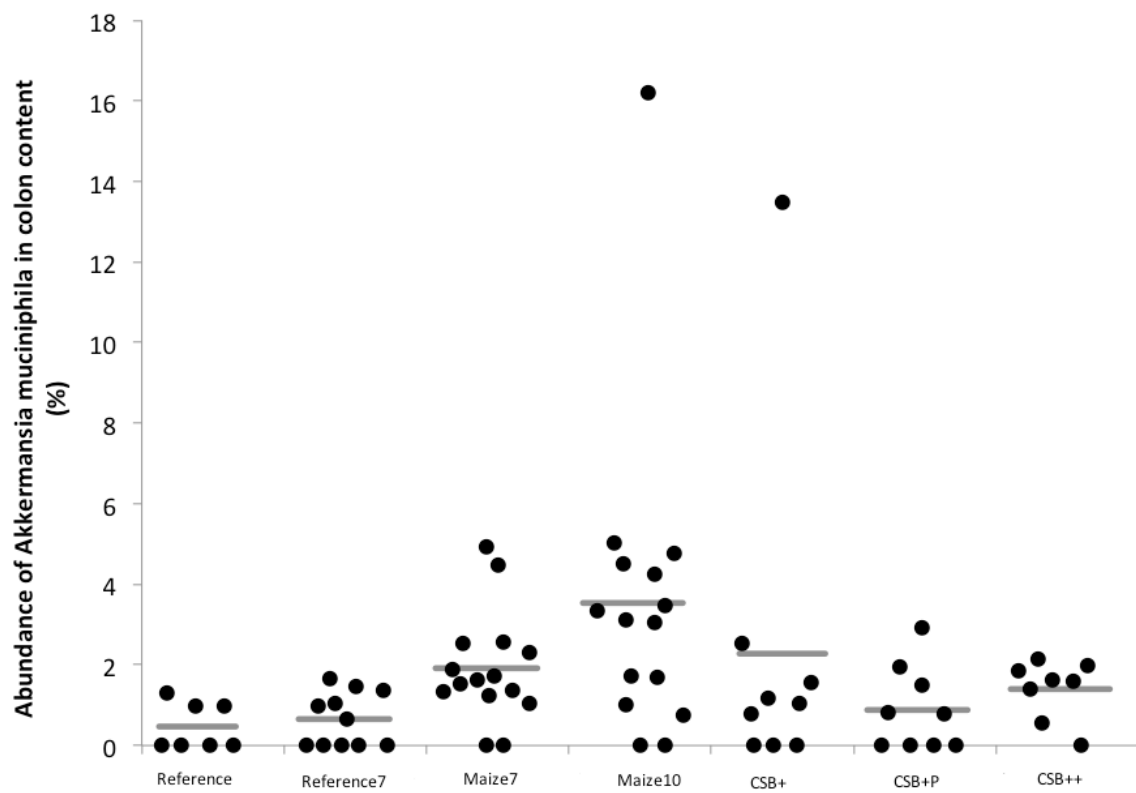

Supplement: Supplementary file 2 — Additional file 2: Figure S2. Relative abundance of Akkermansia muciniphila was determined by qPCR (determined as number of A. muciniphila 16S rRNA gene copies relative to the total number of 16S rRNA gene copies pr. sample). [file 12866_2019_1658_MOESM2_ESM.pdf]

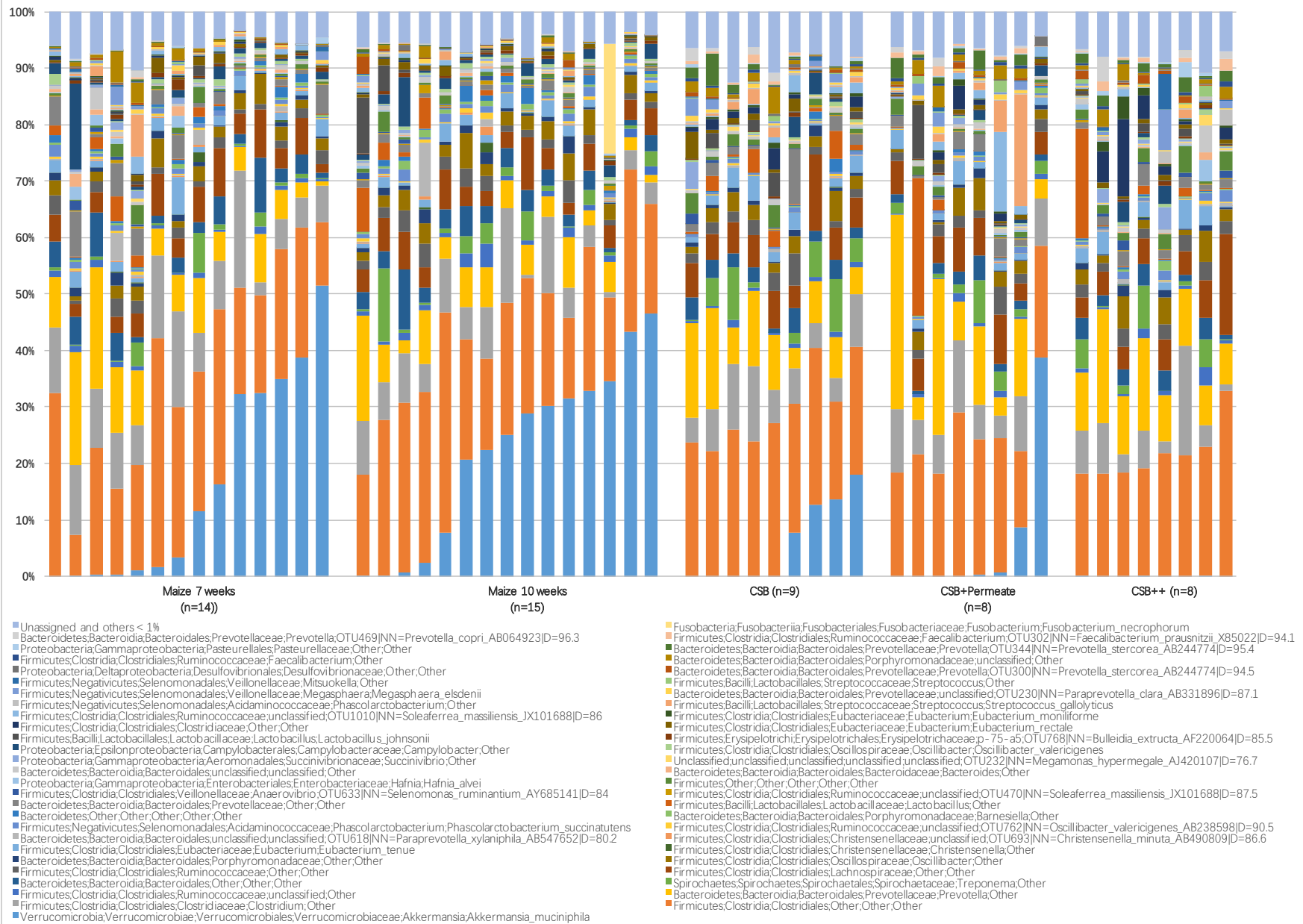

Supplement: Supplementary file 3 — Additional file 3: Figure S3. The relative abundance and distribution of genera detected by targeting V3 V4 region in colon content samples from piglets fed with reference diet for 1 week (reference), then fed with either reference diet or maize for 7 weeks, and lastly fed with either maize, CSB+, CSB + Permeate or CSB++ for 3 weeks. [file 12866_2019_1658_MOESM3_ESM.pdf]

## Slide 1
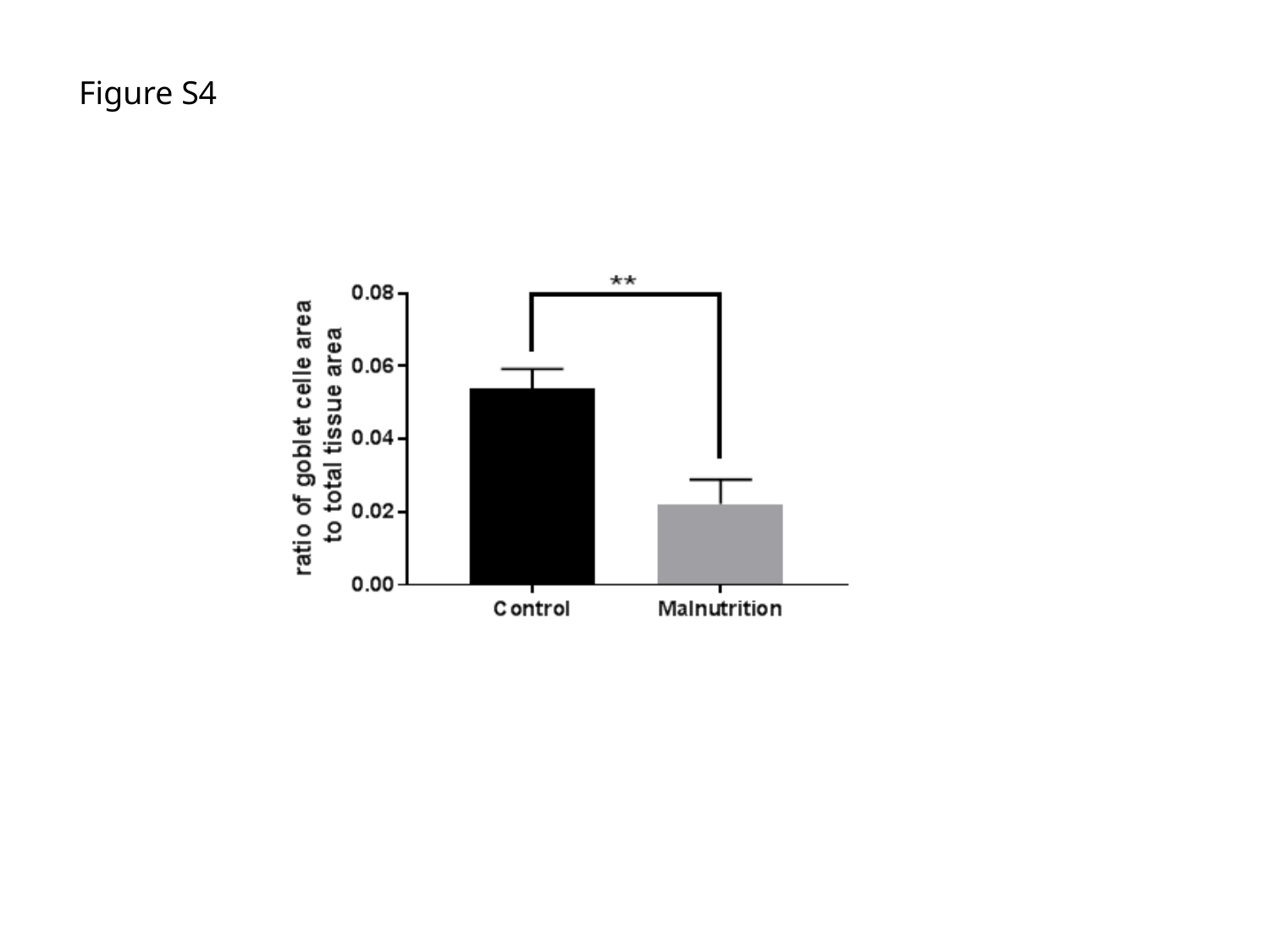

Figure S4

Supplement: Supplementary file 4 — Additional file 4: Figure S4. The ratio of goblet cell area to total tissue area of colon tissue from the reference, well-nourished piglets and the malnourished piglets ere determined by Alcian blue staining. [file 12866_2019_1658_MOESM4_ESM.pptx]
